# MiR-92 Controls Synaptic Development Through Glial Vha55 Regulation

**DOI:** 10.3390/biom15091330

**Published:** 2025-09-18

**Authors:** Simon M. Moe, Alicia Taylor, Alan P. Robertson, David Van Vactor, Elizabeth M. McNeill

**Affiliations:** 1 Program in Neuroscience, Department of Food Science and Human Nutrition, Iowa State University, Ames, IA 50010, USA; moesimon95@gmail.com (S.M.M.); aliciat@iastate.edu (A.T.); 2 Department of Biomedical Sciences, Iowa State University, Ames, IA 50010, USA; alanr@iastate.edu; 3 Department of Cell Biology, Harvard Medical School, Boston, MA 02115, USA

**Keywords:** miR-92, microRNA, drosophila, NMJ, synaptogenesis

## Abstract

MicroRNAs (miRNAs) have emerged as important biomarkers for complex neurological conditions. Modifications in synaptic morphology characterize several of these disease states, indicating a possible role of miRNA in modulating synaptic formation and plasticity. Within the third-instar larvae of Drosophila melanogaster, we uncovered a functional role for highly human-conserved miR-92 in synaptogenesis of the glutamatergic peripheral nervous system. Loss of miR-92 results in underdeveloped synaptic architecture, coinciding with significantly reduced physiological activity. We demonstrate a novel role for miR-92 glial-specific expression to support synaptic growth function and plasticity. Modifications of miR-92 within glial tissue result in aberrant glial barrier properties, including an increased uptake of external dyes. Within the glia, miR-92 regulates a V-ATPase subunit (Vha55), impairing the glial cells from forming appropriate insulating layers around the nervous system. These modifications may impact how the nervous system adapts to its environment, increasing immature ‘ghost bouton’ budding and impairing responses to changes in environmental conditions. Our work highlights the importance of glial-specific miR-92 on synaptic development, affecting glial health and function through its downstream target Vha55, and demonstrates a novel mechanism for glia in synaptogenesis and homeostatic plasticity.

## 1. Introduction

Neurological disease states are complex, resulting from an interplay between biological modifications and environmental effects. Conditions such as Schizophrenia and bipolar disorder have reduced glutamatergic synaptic morphology, hinting at a possible relationship between this morphology and more complex clinical manifestations [1]. The understanding of molecular mechanisms involved in synaptic formation and maintenance has increased dramatically over the past several decades. A relatively novel player in this field are micro-RNAs (miRNAs). These small, non-coding RNAs act as ‘fine tuners’ throughout many species to modulate system-wide homeostasis through post-transcriptional modulation of target messenger RNA (mRNA) [2]. Modulation of neuronal health has been well established for several miRNAs. Within hippocampal neurons, miR-186-5p acts as a negative regulator of GluA2 receptors, facilitating changes in the number of synaptic receptors based on expression [3]. We previously demonstrated that miR-34 activity both presynaptically and postsynaptically at the neuromuscular junction (NMJ) of *Drosophila* can regulate synaptogenesis through a balance of two separate gene targets depending on the tissue compartment [4]. Tissue compartmentalization has also been demonstrated through postsynaptic inhibition of miR-8, resulting in a reduction in NMJ size [5].

Schizophrenia is a neurological condition that has been found to have abnormal levels of circulating miRNA in blood [6]. Among the dysregulated microRNAs is miR-92, a member of a conserved family that includes miR-92a and miR-92b, which share a common seed sequence and are predicted to target overlapping sets of mRNAs [7]. miR-92 is highly conserved in *Drosophila melanogaster* and has been shown to affect memory consolidation in the mushroom body [8]. miR-92 has also been implicated in oncogenesis due to its regulation of genes associated with tumor suppression [9]. Beyond these established roles, recent studies have expanded this miRNA’s role into aspects of motor development, and various health and disease conditions [10,11].

*Drosophila melanogaster* is a good model for experimental manipulation of synaptic formation during development due to its well-characterized peripheral nervous system [12]. Expression of both miR-92a and miR-92b is highest during developmental periods and decreases once the fly reaches adulthood [7]. The conserved excitatory glutamatergic signaling synapses of the *Drosophila* NMJ provide an ideal model for understanding the role of microRNAs on synaptic growth and plasticity [13]. Due to the well-characterized development of the peripheral NMJ, minute changes within the synapse can be observed to elucidate molecular mechanisms of development and maintenance. Genetic tools available within *Drosophila* allow individual gene modulation using spatial and temporal specificity through the Gal4-UAS system [14]. The miRNA-sponge (sp) technology can facilitate competitive inhibition of individual miRNA and, thus, tissue-specific exploration of function [5,14]. This allows us to explore the function of miRNA in the multitude of tissue types surrounding the NMJ to uncover potential roles in synaptic development.

Using both a genetic knockout of miR-92 and miR-sp technology [5,14], we demonstrate that miR-92 contributes to neuronal development of the glutamatergic synapse of *Drosophila* and influences this development within glial cell tissue. miR-92 loss results in overexpression of a V-ATPase subunit (Vha55), leading to a highly permeable glial cell layer and an impairment in synaptogenesis. While the involvement of miRNAs from glia is beginning to emerge, mechanisms by which glial miRNAs regulate synaptogenesis remain relatively unknown. These findings contribute novel insight into prior work in our lab identifying neuronal and postsynaptic tissue regulation of NMJ development [4]. Further, characterizing a potential V-ATPase regulation of glial barrier function expands prior work characterizing glial penetrance. Identifying the role of miRNA within glia is essential for further understanding the relationship between glia and synaptic development. Our research suggests that dysregulation of miR-92 in glia underlies synaptic modifications which may contribute to complex neuronal disease states.

## 2. Materials and Methods

### 2.1. Drosophila Strains and Genetics

All fly stocks were maintained at 25 °C per previously described standard procedures [4]. The miR-sp and Scramble-sp lines were developed by the Van Vactor Laboratory using a pUAST/attB vector with DSRed2 (cloned by Genewiz) with identical insertion sequences to the Generation II miR-sp lines (att-P40; att-P2) [15]. Distinct insertion sites (attP-ZH51C; attP-VK33; Bestgene strain number 24,482 and 9750, respectively) were used to control genetic background with the 20x-sp array in attP40; attP2 sites and mitigate background effects. The miR-92 null line used for the Microarray was from Zhe Han [16]. The miR-92a null and miR-92b lines used in the TEM experiments were a kind gift from the Cohen lab [16]. The miR-92a Null and miR-92b Null lines (published as miR-92a^−/−^ and miR-92b^−/−^) and the miR-92 a/b Null line (published as miR-92^4/4^) were from Fen Biao Gao Laboratory knockout animals and used for the remainder of the experiments [7]. UAS insertion lines and Gal4 lines (Tub-Gal4 (BL5138), Repo-Gal4 (BL7415), and DMef2-Gal4(BL27390)), as well as overexpression lines and their respective control strains (BL 4053, w67C23; BL15018 Kay; BL 20090, Klp68D; BL 16367, CG3408), were obtained from the Bloomington Stock Center (Bloomington, IN, USA). UAS-Vha55 was a gift of the Juan Du and Julian Dau labs. w^1118^ was used as the control for Vha55 line. The following Glial subtype drivers were used: Wrapping glia (Nrv2-Gal4), subperineurial glia (Moody-Gal4), and perineurial glia (C527-Gal4). The glial subtype drivers were a generous gift from the Klämbt lab at the University of Münster. Ok6-Gal4 was a gift from the Griffith lab Brandeis University.

### 2.2. Bioinformatics

Prediction of *Drosophila* miRNA to human gene targets used DIANA Tools version 5.0 [17]. Data mapping between miRbase IDs and miRbase accession was completed using miRBase version 20. Predicted targets were selected through prior indication of a role in NMJ development.

### 2.3. Microarray

Using a mirVana miRNA isolation kit (Invitrogen, Carlsbad, CA, USA), RNA was extracted from 10 pelts (muscle) and 50 ventral nerve chords (CNS) isolated from wandering third-instar larva miR-92 null, as well as Canton S controls. After RNA isolation, Affymetrix *Drosophila* Genome 2.0 Arrays were completed by Expression Analysis-Quintiles Global Laboratories now IQVIA Q2 Solutions. GeneChip scan files were processed with Expression Console v1.4 to produce probe set analysis results using the MAS5 algorithm. Analysis was performed using the Armada algorithm in MATLAB v 7.11.1 as described in [18]. Differentially expressed genes were acquired using parametric (ANOVA, *t*-test), and false discovery rates were controlled using FWER or the FDR with adjusted *p* values (<0.05).

### 2.4. Immunohistochemistry and Quantification of NMJ Development

Wandering third-instar larvae were dissected in a CA^2+^- free saline solution and fixed in fresh 4% paraformaldehyde (vol/vol, Electron Microscopy Sciences, Hatfield, PA, USA) in phosphate-buffered saline for 20 min. Muscle 6/7 motor neuron terminals in abdominal segments A2 and A3 of wandering third-instar larvae were quantified for morphological development of type 1b and 1s glutamatergic boutons. Each experiment’s n value (number of animals) is reported in the figure legends. Animals on average had 2 NMJ measured for an experiment. Analysis of bouton morphology was performed using a Nikon 90i and Nikon Eclipse 80i microscope. Incubation of larvae in primary antibody was performed at 4 °C overnight and for 4 hrs in secondary antibodies at room temperature the following day. The primary antibodies used were as follows: AntiHRP-alexa fluorophore 594 (1:500) (323-585-021 Jackson ImmunoResearch, West Grove, PA, USA), AntiHRP-alexa fluorophore 647 (1:500) (123-605-021 Jackson ImmunoResearch, West Grove, PA, USA), mouse Anti-Bruchpilot (DSHB 1:50) mouse anti-Repo (1:50), mouse Anti-GluRIIA (DSHB 1:10), mouse Anti-Dlg (DSHB 1:50), and rabbit anti-Vha55 (gift from Dr. Julian Dow at the University of Glasgow). The DSHB antibodies developed by The University of California, Berkeley, were obtained from the Developmental Studies Hybridoma Bank, created by the NICHD of the NIH and maintained at The University of Iowa, Department of Biology, Iowa City, IA 52242. Secondary antibodies anti-mouse alexa fluor-488 (Invitrogen, Carlsbad, CA, USA) and anti-rabbit alexa fluor-568 (Invitrogen, Carlsbad, CA, USA) were used at a 1:500 dilution. The larvae were mounted in Slowfade Gold (Thermofisher scientific, Waltham, MA, USA) and stored until imaging at 4 °C. Confocal microscopy was performed using an FV3000 Confocal Laser Scanning Microscope (Olympus, Center Valley, PA, USA). Laser parameters were adjusted to obtain non-saturating conditions prior to acquisition. Data collected was determined to have a normal distribution using Shapiro–Wilk test for normality. For statistical comparisons, a Student’s *t*-test was employed for single comparisons. An ANOVA was employed in experiments that contained several means followed by post hoc *t*-test. Significance is demonstrated using the following parameters: not significant (ns), *p* < 0.05 (*), *p* < 0.01 (**), and *p* < 0.001 (***).

### 2.5. Image Analysis and Quantification

The images were processed and analyzed using ImageJ/Fiji (ver 1.54a). For synaptic bouton quantification. Stacks of 6/7 A2 NMJs were obtained under identical conditions. DLG colocalization with HRP indicated distinct bouton regions. Type 1b and 1s boutons were both quantified. For synaptic architecture quantification at the NMJ (Brp, GluRIIA), masking was performed using HRP to localize structures to the neuronal region, and fluorescence intensity was calculated relative to HRP. Brp punctae were counted and examined relative to the total HRP area from z-stacks. Identification of ghost bouton consisted of the following parameters, HRP+/DLG+ membrane swellings, and Brp-negative. (n = 10–20 animals per genotype).

### 2.6. Electrophysiology

All larval dissections for electrophysiology were conducted as described in Imlach, 2009, using third-instar larvae in Hemolymph-Like saline solution 3.1 (HL 3.1) at a pH of 7.15, containing (in mM) 70 NaCl, 5 KCl, 4 MgCl_2_, 10 NaHCO_3_, 5 Trehalose, 115 Sucrose, and 5 HEPES (300 Osm) [18,19]. Body wall muscles were pinned to stretched positions to optimize electrode placement and recording efficacy at the neuromuscular junction (NMJ). Cell recordings were taken through Clampex version 10.3. Excitatory junctional potentials (EJPs) were recorded intracellularly from the abdominal segments 3–5 muscle at 20–23 °C in 1.5 mM CaCl_2_ Ca^2+^ immediately following mEJP recordings. EJP and mEJP are established, sensitive readouts for NMJ function and homeostatic plasticity. A suction pipette with a tip opening of approximately 10 μm was used to stimulate the segmental nerve (stimulation duration = 0.2 ms, strength = 6 V, rate = 10 Hz). Baseline membrane voltage was recorded for one second prior to stimulation; stimulation was repeated 5 times with 2-s breaks in between stimulations. Cells were discarded if the baseline varied by more than 10 mV during stimulation. Cell recordings between −55 mV and −70 mV were included. All EJP recordings were analyzed in Clampfit version 10.3. Quantal content was calculated dividing the EJP amplitude by the mEJP amplitude. Two-sample *t*-tests of significance were performed between experimental conditions and genotypes (α = 0.05).

### 2.7. Dextran Fluorescence

The following protocol is adapted from Hatan et al., 2011 [20]. Wandering third-instar larvae were placed into a water bath containing 1:50 3 kDa dextran for 30 min. After this, larvae were dissected as outlined earlier to isolate the ventral nerve cord while still attached to the musculature. Tissue fixation was performed without removal of the ventral nerve cord to determine local penetration while still attached to the musculature. Immunofluorescence was performed using mouse anti-FITC to visualize the dextran that penetrated the VNC alongside Anti-HRP-Alexa fluorophore 594 (1:500) for the orientation of neuronal tissue. Fluorescent penetrance was calculated using ImageJ (ver 1.54a), with a correction for background expression with no dextran control (n = 10–20 animals per group).

### 2.8. Potassium Stimulation Assay

The Spaced High K^+^ depolarization Paradigm was followed, as described in Ataman et al., with some modifications to the timing [21]. W^1118^ larvae and miR-92a null third-instar larvae were dissected in normal-HL3 saline containing 0.1 mM Ca^2+^, leaving the CNS and peripheral nerves innervating the body wall muscles intact [22]. Room-temperature high-K^+^ (90 mM) HL3 adjusted for osmolarity changes (in mM; 40 NaCl, 90 KCl, 20 MgCl_2_-6H_2_O, 1.5 CaCl_2_, 10 NaCO_3_, 5 Sucrose, 5 Trehalose, 5 HEPES, pH = 7.2) was applied to unstretched semi-intact preparations with 4 five-minute pulses, each separated by a fifteen-minute incubation in normal-HL3. Experimental preparations were kept in normal-HL3 for 15 min after the last high K^+^ -pulse and then stretched and fixed with 4% paraformaldehyde for 20 min, followed by immunostaining with selected antibodies. Non-stimulated larvae for all experiments were dissected and incubated using the same protocol as above with normal-HL3.

### 2.9. Transmission Electron Microscopy

Dissected peripheral nerve bundles were fixed with 1% paraformaldehyde and 3% glutaraldehyde in 0.1 M sodium cacodylate buffer, pH 7.2 at 4 °C. Samples were washed in the cacodylate buffer 3 times/10 min each and post-fixed with 1% osmium tetroxide in 0.1 M sodium cacodylate buffer for 1 h at room temp. Samples were washed with deionized water 3 times/15 min each and then stained using 2% uranyl acetate in distilled water for 1 h. The samples were washed in distilled water for 10 min and dehydrated through a graded ethanol series (25, 50, 70, 85, 95, 100%) for 1 h each step. Samples were further dehydrated with 3 changes of pure acetone, 15 min each, and infiltrated with EmBed 812 formula (hard) for EPON epoxy resin (Electron Microscopy Sciences, Hatfield, PA, USA) with graded ratios of resin to acetone until fully infiltrated with pure epoxy resin (3:1, 1:1, 1:3, pure) for 6–12 h per step. Nerve bundles on the carapace were placed into beem capsule lids to orient them for cross-sectioning and were polymerized at 70 °C for 48 h. Thick sections (1.5 μm) were made using a Leica UC6 ultramicrotome (Leica Microsystems, Buffalo Grove, IL, USA) and stained with methylene blue and basic fuchsin to identify nerve cross-sections. Thin sections were made at 50 nm and collected onto carbon film grids using a Leica UC6 ultramicrotome (Leica Microsystems Inc., Deerfield, IL, USA). TEM images were collected using a 200 kV JEOL JSM 2100 scanning transmission electron microscope (Japan Electron Optics Laboratories, Peabody, MA, USA) with a GATAN One View 4K camera (Gatan Inc., Pleasanton, CA, USA). Five mages of nerves per animal and 2 animals per genotype were used in this analysis.

### 2.10. Summary of Statistical Analysis

Statistical analyses were performed in GraphPad Prism (version 9.5.1) and R. For pairwise comparisons, unpaired two-tailed *t*-tests were used; for multi-group comparisons, one-way ANOVA with post hoc tests were applied as indicated above.

## 3. Results

### 3.1. Elucidating the Role of miR-92 in Synaptogenesis

**Figure 1:**
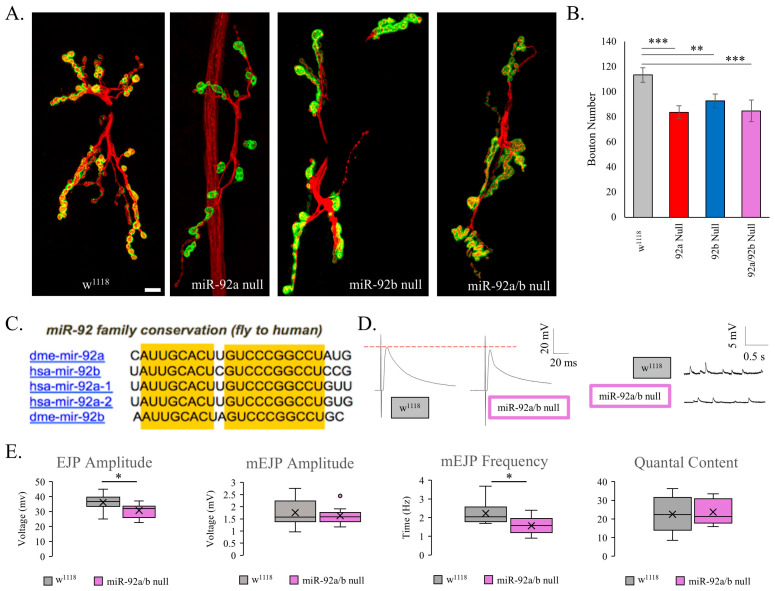
Synaptic development and physiology of mutant miR-92 animals. (**A**) Representative A2 neuromuscular junctions (NMJ) from control flies (w^1118^) and miR-92 null mutant animals. Representative images show HRP in red and DLG in green. Scale bar is 10 μm. (**B**) The total bouton count of type 1a and type 1b synaptic boutons is decreased at the A2 NMJ in miR-92a and miR-92b mutant animals compared to w^1118^. miR-92a/b double mutants, as well as miR-92a and miR-92b mutants, display a significantly reduced number of synaptic boutons compared to w^1118^ flies (*p* < 0.01, *p* < 0.01, *p* < 0.01); n = 11 animals, with 20 NMJs. (**C**) The miR-92 family is highly conserved. Identical seed sequences are indicated in yellow. Dme denotes *Drosophila* miRNA, and hsa denotes human miRNA sequences. (**D**) Representative electrophysiological traces of w^1118^ flies (36 mV) and miR-92a/b double mutant animals (31 mV). EJP and mEJP are shown next to each other, with the red dashed line indicating the peak of the w^1118^ trace; n = 9–10 animals. (**E**) Key characteristics are compared, including EJP amplitude, mEJP amplitude and frequency, and quantal content. miR-92a/b null mutant animals demonstrate a reduction in EJP amplitude (*p* < 0.05), and a reduction in mEJP frequency (*p* < 0.05) compared to w^1118^ animals; n = 9–10 animals. (* <0.05, ** <0.01, *** <0.001).

To understand the role of miR-92 in synaptic development, our lab investigated miR-92 loss and observed the impact on the developing neuromuscular junction (NMJ). Wandering third-instar larvae were quantified for synaptic bouton number at the cleft between muscles 6 and 7 (M6/M7 NMJ) using immunohistochemical markers for presynaptic neuronal membrane, horse-radish peroxidase (HRP), and the postsynaptic scaffold protein, Discs-large (DLG). Null mutant animals for miR-92a, miR-92b, and the double mutant miR-92a/b demonstrate a dramatic reduction in synaptic bouton number (*p* < 0.01, Figure 1A,B). These miR-92 knockout animals have been shown to retain expression of the host gene, jigr [7]. miR-92 has a highly conserved seed sequence between humans and *Drosophila* for both miR-92a and miR-92b (Figure 1C).

Given the gross reductions in synaptic bouton number with miR-92 loss, we researched the synaptic function in these animals. EJP and mEJP are well-established and sensitive readouts for NMJ function and homeostatic plasticity. We performed intracellular electrophysiology on wandering third-instar miR-92a/b null animals to determine if synaptic signaling was altered at these smaller NMJs. Sample traces are shown in Figure 1D. There was a significant decrease in the amplitude of excitatory junction potential (EJP) in miR-92a/b null animals compared to control flies (*p* < 0.05), suggesting that the amount of neurotransmitter filled vesicles and/or the postsynaptic sensitivity was modified in these animals (Figure 1E) [23]. We did not see any change in the amplitude of the miniature events, suggesting that the relationship between the quantity of neurotransmitter contained per vesicle during spontaneous release and the number of postsynaptic glutamate receptors was not altered (Figure 1E) [24,25]. We observed a significant decrease in the frequency of miniature EJP (mEJP) in miR-92a/b null animals (*p* < 0.05), indicating a decrease in the rate of spontaneous release of vesicles from the presynaptic bouton. Quantal content in these animals appeared normal, indicating no significant change to the number of vesicles released in response to an evoked quantal release [23].

### 3.2. Glial-Specific Activity of miR-92

**Figure 2:**
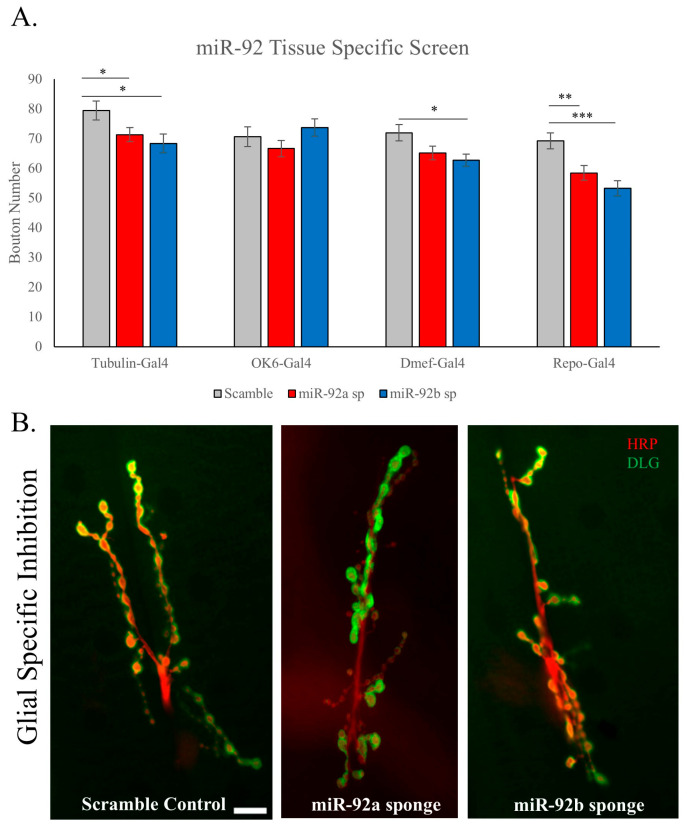
The tissue-specific role of miR-92 in synaptic morphology. (**A**) The total bouton number was quantified after competitive inhibition of miR-92a and miR-92b using a miR-sponge (sp) driven ubiquitously (tubulin-Gal4), in neuronal tissue (OK6-Gal4), in muscle tissue (Dmef-Gal4), and in glial cells (Repo-Gal4). These animals were directly compared to scramble control driven through these same Gal4 drivers to account for any driver effects. miR-92 inhibition through glial tissue resulted in the most significant reduction in bouton number (miR-92a (*p* < 0.01) and miR-92b (*p* < 0.001)); n = 10 animals, 20 NMJs. (**B**) Representative A2 NMJ images of Repo-Gal4-driven inhibition of both miR-92a and miR-92b are shown next to a scramble control to highlight the significant reduction in total synaptic boutons. Scale bar is 10 μm. (* <0.05, ** <0.01, *** <0.001).

To understand the tissue-specific requirement of miR-92 function in the development of the NMJ, we utilized several tissue-specific drivers to selectively inhibit miRNA expression using sp technology. To control for the genetic background, we utilized a scramble control line crossed with the same Gal-4 driver line as the miR-92 sponge. The ‘scramble’ genetic line consisted of 20 random nucleotides not predicted to target any miRNA but allowed for a closer comparison upon tissue-specific upregulation of a target gene allowing us to account for genetic insertion effects. Neuronal specific inhibition (OK6-Gal4) of either miR-92a or miR-92b did not result in a significant difference in synaptic morphology, but muscle-specific inhibition (Dmef2-Gal4) of miR-92b was significantly different (*p* < 0.05) (Figure 2A). Glial-specific inhibition of either miR-92a or miR-92b (Repo-Gal4) resulted in a very dramatic decrease in total bouton counts compared to control flies (*p* < 0.01 and *p* < 0.001, respectively; Figure 2A,B). Our findings parallel those observed in genetic null animals and reinforce a distinct glial-specific role of miR-92, which likely drives the ubiquitous loss phenotype observed (Figure 1B), consistent with established glial contributions to NMJ function [26].

**Figure 3:**
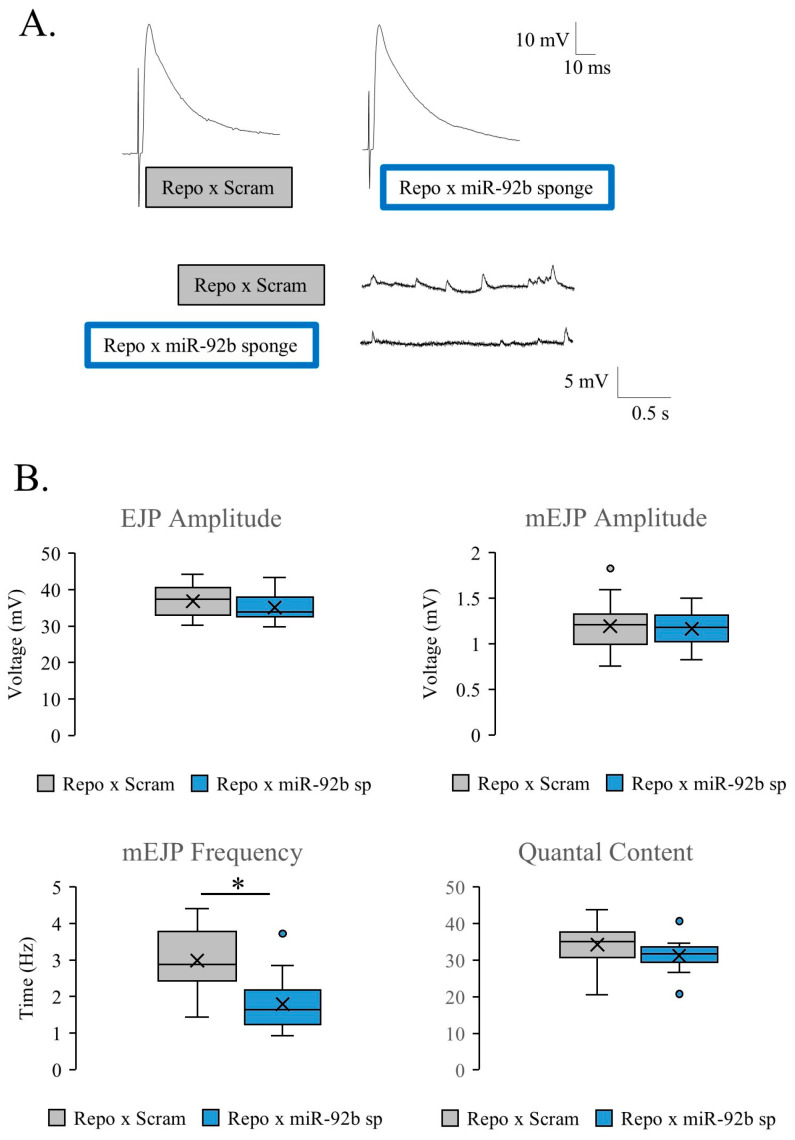
Electrophysiological recordings upon glial-specific miR-92b loss. (**A**) Representative electrophysiology traces of Repo-Gal4-driven loss of miR-92b are shown compared to animals expressing scramble through Repo-Gal4. EJP and mEJP are both included; n = 10–11 animals. (**B**) Key electrophysiological parameters are indicated, including EJP amplitude, mEJP amplitude and frequency, and quantal content. miR-92b inhibited by Repo-Gal4 resulted in a significant reduction in mEJP frequency compared to scramble controls, supporting a functional role for miR-92 in glia (*p* < 0.05); n = 12–15. (* <0.05).

To determine the contribution of glial-specific miR-92 inhibition to physiological changes observed in the null animals, we used Pan-glial (Repo-Gal4)-driven miR-92b sponge animals. We assayed electrophysiological activity in wandering third-instar larvae. Sample traces are shown in Figure 3A. We did not see a change in the EJP between mir-92b sponge animals and healthy controls (Figure 3B), which is different from the miR-92 a/b double null mutants. Consistent with the double mutant, we found there was no significant difference in the mEJP amplitude, but we did find a dramatic decrease in mEJP frequency in miR-92b sponge animals compared to scramble control animals (*p* < 0.01) (Figure 3B).

### 3.3. Synaptic Machinery and Ultrastructure in miR-92 Loss

**Figure 4:**
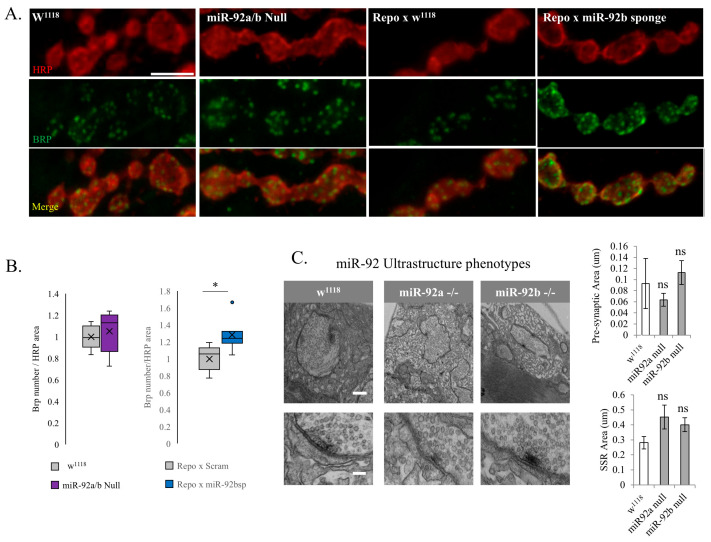
The impact of miR-92 loss on synaptic machinery and ultrastructure. (**A**) Representative immunoreactivity in a terminal region of the A2 NMJ of w^1118^, miR-92a/b null animals, Repo-Gal4 crossed with w^1118^, and Repo-Gal4 crossed with miR-92b sponge. HRP is represented in the top panels (red). Scale bar is 5 μm. Brp is indicated in the middle panels (green), and the bottom panels of A are the merged images. (**B**) The number of Brp punctae trends higher in miR-92a/b null mutants but is not significant (left panel); a significant increase in Brp number is observed in Repo-Gal4-driven miR-92 sponge animals relative to Repo-Gal4-driven scramble controls (*p* < 0.05); n = 7 animals. (**C**) Representative TEM of miR-92a and miR-92b null mutant animals (n = 3 animals per group and 7 boutons per animal). Representative images of cross-sections of synaptic boutons (n = 7 boutons per animal) and zoomed-in images showing clustering of synaptic vesicles near active zones, indicated by electron dense structures in the lower images. Quantification of presynaptic area and postsynaptic SSR area indicate no significant difference in either miR-92a or miR-92b null animals. Scale bar for top panels is 500 nm and bottom panel is 5 μm. (not significant (ns), * <0.05).

To examine the synaptic release machinery, we utilized an immunohistology marker for Bruchpilot (Brp), a protein necessary for synaptic coupling of vesicles before release. Although no change was found in miR-92a/b null mutant animals, a significant increase in Brp number relative to the HRP area was present in Pan-glial (Repo-Gal4)-driven miR-92b sponge animals compared to their control (Figure 4A,B). These modifications in presynaptic architecture could be a homeostatic response to the decreased synaptic bouton count, indicating that individual boutons acquired greater numbers of Brp for synaptic release in a manner that did not depend on increasing the number of boutons. The increase in Brp observed in the miR-92b sponge animals could lead to the relatively normal EJP observed in these animals. To characterize the glutamate receptors, an antibody for GluRIIA was used, and fluorescence intensity was measured, however both miR-92a/b null mutant animals and Pan-glial (Repo-Gal4)-driven miR-92b sponge animals were unchanged relative to control (Supplemental Figure S1A,B respectively).

We utilized transmission electron microscopy (TEM) and found no significant difference in the presynaptic bouton area of miR-92a or miR-92b mutant flies compared to control flies (Figure 4C). Additionally, there were no changes in the subsynaptic reticulum (SSR) area surrounding boutons of the miR-92 mutant animals compared to control flies. The active zones in the miR92a^−/−^ and miR92b^−/−^ animals were intact, with no significant differences observed relative to control animals, supporting and overall normal morphology of the synapse.

### 3.4. Targets of miR-92

**Figure 5:**
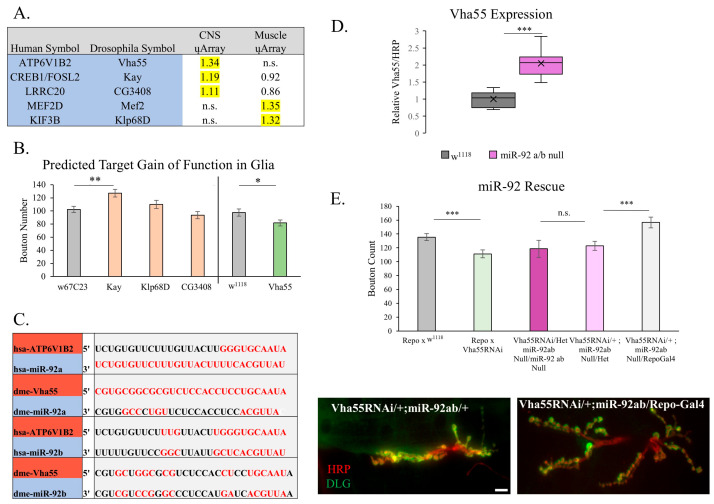
Phenotypic characterization of putative miR-92 targets with expression changes in miR-92 loss and validation of Vha55 as relevant target. (**A**) The top 5 candidate targets of miR-92 based upon seed sequence, NMJ function, and human homology are indicated. Respective symbols for humans and *Drosophila* are shown. Yellow is used to indicate microarrays showing values over 1 in either CNS or muscle, showing expression at the NMJ. (**B**) The five predicted targets were overexpressed using Repo-Gal4 and total bouton counts at the NMJ. Vha55 is the only target to replicate a significant decrease in the number of synaptic boutons upon pan-glial-specific (Repo-Gal4) overexpression (*p* < 0.05). w67C23 is the control for the Kay, Klp68D, and CG3408 overexpression lines, and w^1118^ was used as the control for Vha55, given the specific insertion background of the respective overexpression lines. Overexpression of Dmef2 was embryonic lethal, so further data is not included; n = 10–11 animals, with 20 NMJs. (**C**) Sequence homology of Vha55 and its human equivalent, ATP6V1B2, with the respective *Drosophila* and human miR-92 sequences. (**D**) Fluorescence intensity of Vha55 was quantified within miR-92a/b null mutants using an antibody conjugated against Vha55, using HRP as a relative fluorescent control. There was a significant increase in relative Vha55 immunoreactivity compared to control animals at the NMJ (*p* < 0.001); n = 14–18. (**E**) Rescue crosses in a miR-92 a/b null mutant background overexpressing Vha55 RNAi with Pan-glial driver (Repo-Gal4) demonstrates a significant rescue in bouton number back to control levels (*p* < 0.001). Representative NMJs are shown to demonstrate the return to normal synaptic bouton numbers upon rescue. Scale bar is 10 μm; n = 10–12 animals. (not significant (n.s.), * <0.05, ** <0.01, *** <0.001).

We utilized the high degree of human conservation of miR-92a and miR-92b to identify and prioritize predicted mRNA targets underlying the morphological changes observed at the NMJ. An initial target analysis was performed to identify 349 in silico predicted targets using the seed sequence of miR-92. To narrow the focus to relevant genes, we triangulated those genes that had (1.) prior evidence of function at the NMJ, (2.) conserved miRNA Response Element (MRE) in humans, and (3.) a significant increase in expression in microarray analysis of pooled tissue from dissected larval CNS or larval muscle pelt. From this prioritization, five targets emerged. These five candidate genes were Vacuolar H^+^-ATPase 55kd subunit (Vha55), Kayak (Kay), CG3408, myocyte enhancer factor 2 (Mef2), and Kinesin-like protein at 68D (Klp68D) (Figure 5A). The full microarray can be found in Supplemental Table S1.

These five predicted targets of miR-92 were overexpressed using the Pan-glial driver Repo-Gal4. The overexpression lines Kay, Klp68D, CG3408, and VHA55 were each crossed with Repo-Gal4 and compared to genetic background controls (w67c23 and w^1118^, respectively) also crossed with Repo-Gal4. Quantification of synaptic bouton number was performed as outlined previously. Figure 5B demonstrates that overexpression of Klp68D and CG3408 did not significantly change the total bouton count, a neutral result when compared to miR-92 loss. In contrast, overexpression of Kay increased with bouton number (*p* < 0.01), a negative result when compared to miR-92 loss. Overexpression of Mef2 was embryonic lethal, so we could not quantify any NMJ changes at the third-instar larvae stage of development. Vha55 glial overexpression resulted in fewer synaptic boutons at the NMJ, mimicking the effect we observed with glial-specific inhibition of miR-92b (*p* < 0.05). Among our five potential targets, Vha55 was the sole candidate that phenocopied miR-92 loss of bouton number.

Sequence homology of Vha55 and human counterpart is shown relative to both *Drosophila* and human miR-92 seed sequences (Figure 5C). To determine if the level of Vha55 was modified upon loss of miR-92, we measured the fluorescence intensity of Vha55 proteins at the NMJ in miR-92a/b null mutant animals using a primary antibody raised against Vha55. We found that there was a significant increase in the amount of Vha55 protein at the NMJ in these animals (*p* < 0.05) (Figure 5D). We validated this antibody using a Vha55-RNAi knockdown in the glia (Supplemental Figure S1C). To confirm the specificity of Vha55 as a target of miR-92 in the development of the synaptic boutons, we overexpressed a Vha55-RNAi line in the miR-92a/b null background. Expression of the Vha55-RNAi in the miR-92a/b null background resulted in the synaptic bouton counts to be rescued to control levels, supporting the idea that Vha55 is the relevant downstream target of miR-92 in synaptogenesis (Figure 5E). We did not see any significant change in Brp and GluRIIA expression, indicating that miR-92 likely has additional targets contributing to these observed phenotypes at the synapse (Supplemental Figure S2).

### 3.5. Disruption of Glial Barrier Capabilities

**Figure 6:**
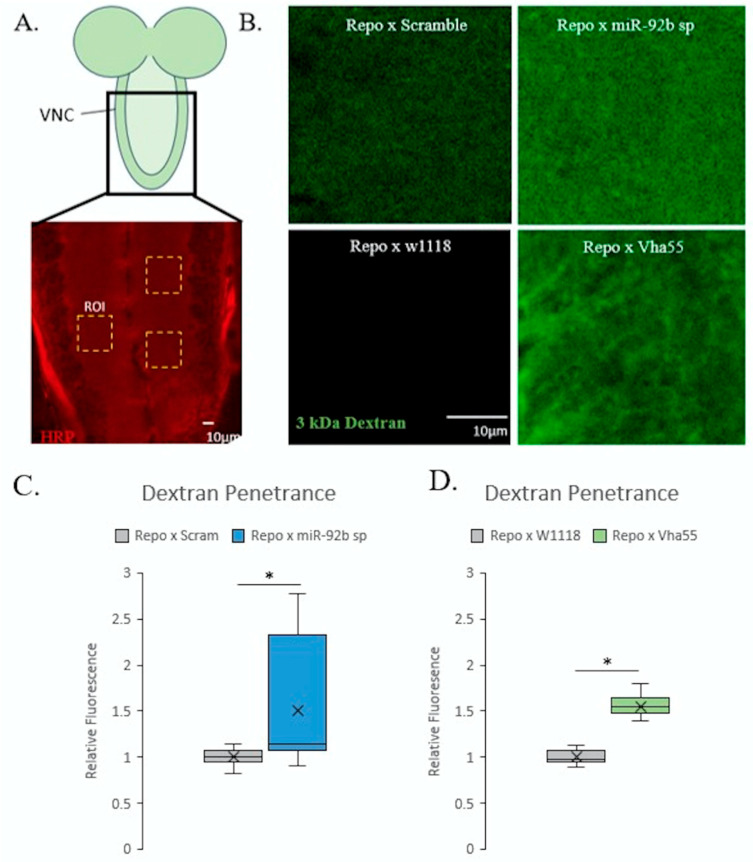
Impaired blood–brain barrier capabilities upon miR-92 loss in the ventral nerve cord (VNC). (**A**) Fluorescence intensity inside the VNC as visualized through dextran dye penetration. Top panel, cartoon of neuropil where total relative fluorescence intensity of 3 regions of interest (gold boxes indicated in HRP-stained wild-type neuropil, bottom panel) were randomly selected and averaged for each animal; n = 8. (**B**) Representative regions of interest (gold boxes) from Repo-Gal4 driving the miR-92b sponge, and Repo-Gal4 driving Vha55 overexpression compared to respective control animals (left panels); n = 7–11. (**C**) Quantification of dextran fluorescence intensity in Repo-Gal4 driving miR-92b loss relative to HRP. (**D**) Quantification of dextran fluorescence intensity in Repo-Gal4 driven Vha55 overexpression relative to HRP. The results show increases in fluorescence in miR-92b sponge animals (*p* < 0.05) and Vha55 overexpression (*p* < 0.05) compared to respective controls; n = 12 animals. (* <0.05).

Several glial tissues encompass motor axons and synaptic terminals, including the wrapping glia (WG), subperineurial glia (SPG), and perineurial glia (PG). Each of these glial cells performs vital functions necessary for the appropriate development and function of the NMJ. Since miR-92 demonstrated a significant role within glial cells, we performed several assays to determine if the function of glial cells was impaired. We measured the function of the SPG, which is responsible for insulating the nervous system from the K^+^-rich hemolymph [26,27]. The introduction of 3 kDa fluorescent dextran dye was used to determine the insulating capacity of the SPG. A prior protocol assessing the health of SPG through the level of dextran dye penetrating the larvae was adapted for use in this study [20]. Larvae were submerged in dextran dye and dissected to determine the fluorescence intensity that could penetrate the SPG. Figure 6A, specifically the gold boxes, indicates where measurements of fluorescence were taken from the ventral nerve cord, as well as showing representative roi images. Glial (Repo-Gal4)-driven loss of miR-92b resulted in a less dramatic but significant increase in dextran penetrance (*p* < 0.05) compared to control flies (Figure 6B,C). Furthermore, we observed a significant increase in dextran dye that was able to penetrate the ventral nerve cord (VNC) of glial-specific (Repo-Gal4)-driven Vha55 overexpression animals (*p* < 0.001), indicating a possible reduction in barrier function (Figure 6D).

### 3.6. miR-92 Glial Subtype Specificity

**Figure 7:**
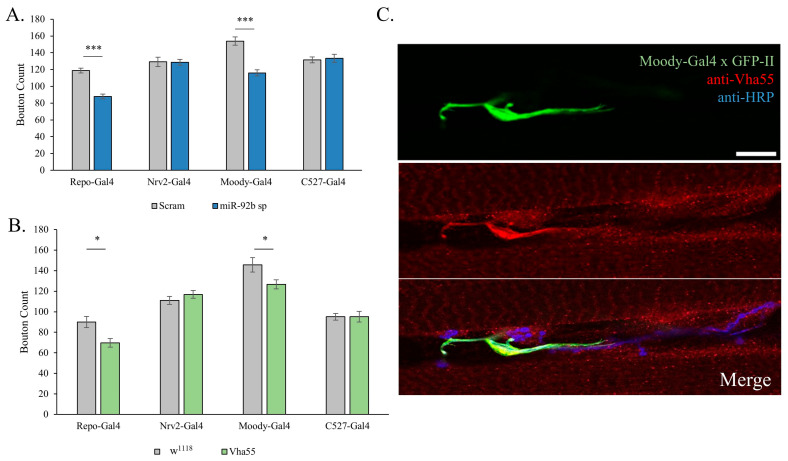
miR-92 and Vha55 function in the subperineurial glia. (**A**) Glial cell subtype-specific drivers were crossed to the miR-92b sponge. To control for Gal4-driver effects, each driver was crossed with a scramble control (gray bar) and compared to miR-92b sponge specific loss (blue bar). Similarly to Repo-Gal4 inhibition, SPG-specific loss (Moody-Gal4) resulted in significant reductions in bouton count (*p* < 0.001); n = 10–12. (**B**) Overexpression of Vha55 within glial cell subtypes. Overexpression of Vha55 through subperineurial glia results in a significant reduction in bouton count, mimicking loss of miR-92b (*p* < 0.05); n = 10–13 animals, with 20 NMJs. (**C**) Fluorescent immunostaining using rabbit anti-Vha55 at the NMJ colocalized with a GFP reporter driven by Moody-Gal4, indicating expression of Vha55 at the NMJ is localized in the SPG n = 6 animals. Scale bar is 10 μm. (* <0.05, *** <0.001).

To determine if there was a specific glial cell type where miR-92 activity was required for NMJ development, we used glial subtype-specific drivers for the three glial subtypes found at the NMJ, WG (Nrv2-Gal4), SPG (Moody-Gal4), and PG (C527-Gal4). Each of these were crossed to miR-92b sponge animals and quantified using immunofluorescence. We found that inhibition of miR-92b in the SPG recapitulated the loss of total boutons observed from the Repo-Gal4 cross (*p* < 0.001, respectively) (Figure 7A). Inhibition of miR-92b through WG and PG did not significantly change synaptic bouton count.

We examined the role of Vha55 modulating the glial subtypes. Overexpression of Vha55 within WG and PG did not result in significant changes in synaptic bouton number. Overexpression in the SPG significantly decreased the total bouton count (*p* < 0.05, Figure 7B). Immunofluorescence of Vha55 at the NMJ illustrates the significant overlap between a GFP-reporter driven in the SPG and anti-Vha55 (Figure 7C). This supports the idea that Vha55 is the relevant miR-92 target in the SPG driving NMJ morphology.

Due to the observed changes in SPG function, we utilized TEM and examined the septate junction in miR-92 loss. Supplemental Figure S3A shows the characteristic ladder-like structure of the septate junction in the Repo-Gal4 x w^1118^ and w^1118^ micrographs. We observed that, compared to control animals, loss of miR-92a/b or overexpression of Vha55 in glia resulted in regions of septate junction that lacked well-defined structures (indicated by white brackets in the figure). Although the overall glial structure was largely intact in the cross-sections of these axons, we also observed an increase in thickness of the neural lamella compared to control animals (Supplemental Figure S3B, white brackets). These observations suggest the development of the subperineurial glia may be impaired.

### 3.7. Modifications in Bouton Addition

**Figure 8:**
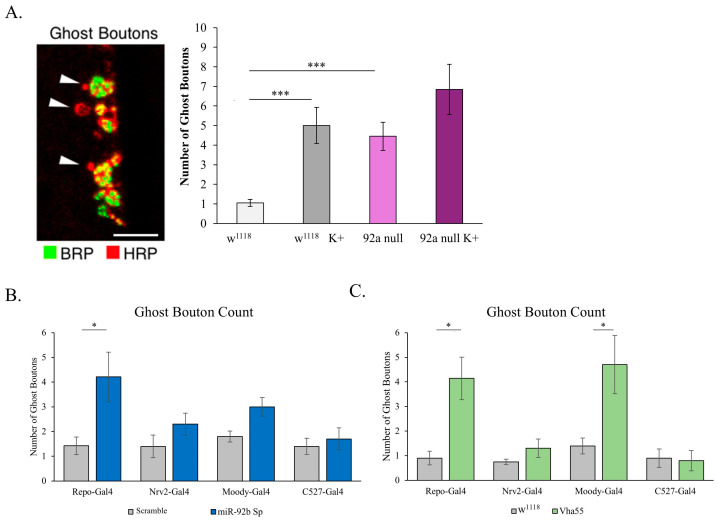
Increased ghost bouton formation upon both miR-92 and Vha55 modification. (**A**) The addition of ghost boutons lacking Brp occurs in w^1118^ animals upon addition of high K^+^-rich hemolymph (*p* < 0.001). miR-92a null mutant animals display an increased level of ghost boutons at baseline without exposure to K^+^-rich hemolymph (*p* < 0.001) and do not significantly increase their ghost bouton number upon stimulation. Scale bar = 5 μm; n = 10 animals, with 20 NMJs. (**B**) Baseline ghost boutons were measured upon miR-92b sponge inhibition driven through various glial subtype specific drivers. Pan-glial (Repo-Gal4) miR-92b sponge animals have a significantly increased number of ghost boutons (*p* < 0.05) compared to control flies; n = 10 animals, with 20 NMJs. (**C**) Ghost boutons were measured upon glial subtype overexpression of Vha55. Pan-glial (Repo-Gal4) Vha55 overexpression resulted in a significant increase in the number of ghost boutons (*p* < 0.05). Vha55 driven through subperineurial glia (Moody-Gal4) also resulted in a significant increase in the number of ghost boutons (*p* < 0.05); n = 10 animals, with 20 NMJs. (* <0.05, *** <0.001).

The SPG tightly regulates the movement of ions and larger molecules both into and out of the nervous system. One of the molecules it regulates is K^+^ (potassium). Potassium stimulation of the NMJ challenges the nervous system to homeostatically scale by increasing the number of ghost boutons, and this process is primarily thought to take place through increased synaptic activity [21]. These immature boutons lack synaptic release machinery (BRP), as has been shown through immunofluorescence [28]. To test how loss of miR-92 affected synaptic maturation, we stimulated miR-92 null animals with high concentrations of K+, and normal homeostatic scaling response was not observed (Figure 8A). Further, at baseline, there is a greater number of ghost boutons in miR-92 mutant animals, suggesting that miR-92 activity is important for synaptic maturation. Due to ghost boutons existing as a normal stage of bouton development, a few ghost boutons are expected in a healthy NMJ. However, the high number in mutants may indicate that synaptic development pauses during the addition of new boutons, leaving more boutons in the immature state. Upon Pan-glial (Repo-Gal4)-driven inhibition of miR-92b, we see a similar increase in ghost bouton counts (*p* < 0.05) (Figure 8B). However, the loss of miR-92b does not significantly increase ghost boutons in any of the glial subtype drivers. This suggests that miR-92 activity may be required in more than one glial subtype.

Further assessment of ghost bouton formation upon overexpression of Vha55 in glia confirmed that Pan-glial (Repo-Gal4) overexpression of Vha55 resulted in a similar increase in the number of ghost boutons as observed in miR-92 loss-of-function animals (*p* < 0.05) (Figure 8C). Interestingly, overexpression of Vha55 through SPG resulted in a significant increase in ghost bouton formation (*p* < 0.05), similar to that seen through the Pan-glial driver (Figure 8C). This result further reveals Vha55′s role within SPG to mediate synaptic growth.

Our data supports a requirement for miR-92 in glia to properly develop synaptic boutons through its target Vha55. This highlights the importance of highly human conserved miR-92 within glia, and its impact on the closely associated motor neuron development at the NMJ. This research demonstrates that glial-derived miRNAs can play a key role in the tight regulation of synaptic architecture in developing *Drosophila*.

## 4. Discussion

### 4.1. Glial Involvement in Synaptic Development

Growing evidence indicates a significant role for miRNAs in neuronal health. However, many of these miRNA have yet to be characterized, and much remains unknown regarding how individual miRNAs directly impact neuronal development in disease, as reviewed in Chen and Chen [29]. Here, we demonstrate that highly human conserved miR-92 is required in glia for appropriate neuronal development and function of the larval peripheral NMJ. Within *Drosophila*, the development of the NMJ and its glial components are closely related and well characterized [28]. Development of both perineurial and subperineurial glia has been shown to match motor neuron development as the *Drosophila* larvae develops [30]. Kerr et al. found that the glia surrounding the NMJ secrete wingless (wg) to modulate glutamate receptor clustering and, thus, physiological properties [31]. Glia have many diverse roles within the nervous system of *Drosophila*, both insulating the nervous tissue and regulating neurotransmitter levels in adult muscles around synaptic connections [32,33].

The specific involvement of miRNA within glia has yet to be well explored. Human miR-125 was shown to promote gliogenesis within astrocytes in response to Interleukin-6 stress [34]. It has also been demonstrated previously by Prada et al. that in vitro inflammatory microglia transfer miR-146 by extracellular vesicles to decrease the spine density of dendrites [35]. Prior research in *Drosophila* has demonstrated tissue-type specific miRNA regulation of NMJ development. McNeill et al. demonstrated that miR-34 mediates neuronal development through both presynaptic Nrx-IV and postsynaptic Hts [4]. Additionally, miR-274 has been found to be secreted from peripheral glia to modulate synaptic bouton and tracheal branch growth [36]. Our work on miR-92 compliments this work, further demonstrating the importance of glial miRNAs.

### 4.2. miR-92 Regulation of NMJ Morphology and Function

One of the more prominent attributes of the *Drosophila* synapse is its plasticity. Reductions in bouton number in both miR-92a/b null and Pan-glial (Repo-Gal4) miR-92b suppression results in physiological defects. Reductions in spontaneous mEJP events indicate that loss of miR-92 in glia leads to fewer spontaneous vesicle fusion events. In both scenarios, there appears to be an increase in Brp punctae, possibly to offset and return the resulting release sites to a more appropriate level. Studies have demonstrated that only 25% of active zones at the *Drosophila* NMJ contribute to distinctly spontaneous release, possibly lacking presynaptic Cac channels to facilitate Ca^2+^ influx [37]. The remainder of the active zones specialize in distinct electrical signaling. Clustering of active zones also appears important, as there is a preference for clustering near high-volume receptor postsynaptic membranes [37]. It is important to note that these Brp modifications seen in our animals do not correspond to changes in total bouton size, indicating that synaptic release machinery is being modified outside of mechanisms that change global neural tissue size. The dramatic decrease in synaptic activity seen in the miR-92 a/b null mutant animals could be due to the activity of miR-92 in a tissue outside of glia facilitating structures required for appropriate physiology. A decrease in the total quantity of boutons has been shown to not directly lead to changes in electrophysiology data [38]. It has been demonstrated that global inhibition of miR-92 in adult flies reduces average speed, acceleration, and shorter travel distance when compared to controls [10]. Further research is necessary to explore if there are any modifications in gross behavior upon glial-specific loss of miR-92b. Additional experiments could provide insight into specific physiological effects arising from loss of miR-92. Given the strong, convergent morphological effects upon loss of miR-92 (NMJ underdevelopment, increased ghost boutons), alongside barrier deficits, our electrophysiological findings support our claims of miR-92 requirements for appropriate synaptic signaling.

### 4.3. Potential Targets of miR-92

In silico target prediction for miR-92 resulted in many potential target genes. Only two of these targets, upon overexpression, resulted in modifications in synaptic bouton number. These two targets were Kay and Vha55. Kay is the *Drosophila* homolog of fos and forms the transcription factor AP-1 through the heterodimerization of both it and Jun [39]. Gain-of-function of Kay and, thus, induction of AP-1 leads to increased neurotransmitter release at the *Drosophila* larval NMJ through movement of the vesicle pools from the reserve pool to the readily releasable pool (RRP) [40]. Previous exploration of AP-1 has indicated that inhibition resulted in lower dendritic growth of motor neurons, highlighting a possible relationship between Kay and NMJ development [41]. As overexpression of Kay presented an inverse phenotype to what we observed upon loss of miR-92, we focused on further exploration of Vha55.

In addition to an upregulation observed in the null mutant animals at the NMJ, we found many key similarities to phenotypes observed in the loss of miR-92 with Vha55 overexpression. Further, the knockdown of Vha55 in glial cells was able to rescue bouton number phenotypes in miR-92 loss. V-ATPases acidify endocytic compartments and regulate intracellular transport [42]. Vha55 has demonstrated roles in embryonic development, but very little work examining its direct relationship within the glia at the NMJ has been performed [42,43]. In the human equivalent, ATPV1B2, specific single-nucleotide polymorphisms (SNPs) have been associated with lifetime occurrences of depression, suggesting V-ATPase activity is related to general neuronal well-being and cognitive impairment [44]. Vha55 is a subunit of the larger Vacuolar ATPase proton pump that mediates membrane trafficking within *Drosophila*, and is essential for function [43,45]. Their primary function is to acidify either endosomes or lysosomes [46]. The V1 subunit of the Vacuolar ATPase sits in the cytosol and hydrolyzes ATP into ADP and phosphate, creating a torque movement that facilitates the movement of protons into the lumen. Vha55 is within this V1 subunit. Importantly, our microarray data indicate that other components in the Vacuolar ATPase complex, in addition to Vha55, are coordinately upregulated by loss of miR-92b activity. Several additional predicted targets were also identified, spanning pathways involved in vesicle trafficking, cytoskeletal regulation, and signaling networks relevant to barrier and synaptic function. While these were not extensively validated in the present study, they represent strong candidates for future investigation and suggest that miR-92 may regulate a broader transcriptional program within glial cells.

Given the scope of predicted interactions, transcriptomic and biochemical approaches will be valuable next steps to expand the target landscape. Small RNA-seq profiling could define the full spectrum of miR-92–dependent gene expression changes in glia, while CLIP-based approaches (e.g., HITS-CLIP, PAR-CLIP) would enable identification of direct miR-92–mRNA binding interactions. Integration of these methods with functional assays at the NMJ and BBB could illuminate whether miR-92 coordinates a multi-gene program regulating synaptic growth, vesicle recycling, and barrier stability. Vha55 expression is vital, as the null *Drosophila* mutant is embryonic lethal [43]. These same mutants demonstrate a significant reduction in lysosomal function, while embryos, as indicated by prior analysis, show an increased build-up of lysosomal cargo and an increase in multivesicular bodies [47]. The lysosome is instrumental within cells for the sorting and degradation of extracellular proteins and components [48]. Our data demonstrate that Vha55 loss alone also results in a significant decrease in bouton number, supporting the importance of tight regulation of its expression. Our work specifically focused on neuronal regulation and development through glial-specific miR-92. Future work is needed to identify whether the additional targets identified in our microarray function downstream of miR-92.

### 4.4. Involvement of the Subperineurial Glia

The similarity of phenotypes found between miR-92 null mutants and glial-inhibited miR-92b animals suggests that glial expression of miR-92 is the origin of influence on the development of the NMJ. The decreased expression of the glial-specific transcription factor Repo in miR-92 mutant animals indicates compromised glial health with loss of miR-92, suggesting that miR-92 expression may influence glial cell development [49]. Aged miR-92 a/b double null animals have smaller total brain sizes than control animals with fewer total neurons and glial cells [7]. This was hypothesized to result from reduced proliferation and premature differentiation of neuroblasts. In our studies, we can identify relatively normal glial morphology in peripheral axons, indicating that this cell population is not grossly affected. Future work could utilize time course- and dose-specific experiments to explore how the mechanistic effect downstream of glial-specific miR-92 affects both development and aging.

As our work demonstrates, loss of miR-92 within the confines of the NMJ appears to result in the strongest phenotypes when expression is restricted within a specific subset of glial cells. Comparing phenotypic changes that occurred with loss of miR-92b and with overexpression of Vha55, it appears that the SPG is where miR-92b expression plays a key functional role. A decrease in total boutons that mirrored global glial loss was found by inhibiting miR-92b within the SPG. However, while SPG-specific inhibition of miR-92b reduced total bouton number to a degree comparable with pan-glial loss, it did not significantly alter ghost bouton number. This divergence suggests that bouton formation and bouton maturation are regulated by distinct glial contributions: the SPG plays a dominant role in establishing bouton number, but additional glial subtypes are likely required to coordinate bouton maturation and prevent ghost bouton accumulation. Thus, the ghost bouton phenotype observed with pan-glial inhibition probably reflects the combined loss of miR-92 activity across multiple glial subtypes, rather than SPG alone. The SPG makes up the blood–brain barrier (BBB) in *Drosophila*, insulating its peripheral nerves and CNS from the high-potassium hemolymph and mediating transport into and out of the nervous system [50]. Total loss of function of this glial subtype has been shown to dramatically reduce larval crawling patterns and amplitude of the EJP, suggesting we are likely seeing impairments of its function instead of a total loss of this cell type [51]. If loss of miR-92 activity leads to a reduction in SPG function, then this could contribute to the reduction in synaptic activity observed in miR-92 mutant animals.

The SPG cells comprising the BBB are joined by protein complexes termed septate junctions, which seal the cellular layer and protect against paracellular transport [49]. Proper endosomal signaling is required for junction development; for example, Neuroglian colocalizes with early and sorting endosomes through the action of an iron-binding epithelial protein that mediates junctional protein endocytosis [52]. Because this trafficking occurs during embryogenesis, disruptions at this stage may impair junction formation. Supporting this, Vha55 LOF studies show its expression is required for survival beyond embryogenesis [43]. In our study, Vha55 colocalized with SPG at the NMJ, and its glial overexpression increased BBB permeability to dextran, phenocopying miR-92a/b null and glial miR-92b sponge animals. These findings suggest that Vha55 regulates septate junction formation and stability, as glial-specific overexpression produced junctional discontinuities.

Genetic loss of Moody, a GPCR expressed in SPG, similarly increases dextran penetrance [20]. Moody mutants also exhibit disrupted septate junctions and swollen nerves [53], and NrxIV knockdown in SPG results in locomotor defects, swollen nerves, and Brp accumulation [54]. Because our flies did not display swollen nerves, this suggests that miR-92–modulated Vha55 may regulate barrier penetrance independently of Moody. Consistently, we observed increased Brp punctae within boutons and alterations in septate junction morphology. The elevated dextran uptake we report across multiple genetic conditions may, therefore, reflect impaired junction assembly and function arising from aberrant Vha55 activity. Together, these results build upon prior studies of barrier regulation and reveal a potential novel link between V-ATPase function and septate junction development mediated by glial-specific miR-92.

Loss of the septate junction protein NrxIV dramatically reduces evoked responses [54]. In contrast, Vha55 overexpression did not cause a comparable reduction, suggesting that while BBB permeability is increased by Vha55 modulation, the barrier and its associated proteins are not completely compromised. Schmidt also reported reduced larval crawling in NrxIV mutants [51]. Locomotor roles of miR-92b have likewise been studied: neuronal loss increased larval speed and distance traveled, whereas ubiquitous loss reduced adult speed, bout number, and distance [10]. Whether glial-specific loss of miR-92 or elevated Vha55 similarly affects locomotion remains to be determined and would clarify how barrier dysfunction relates to neural circuit output [31]. Importantly, barrier impairment does not always correlate with overt structural changes: Delta mutants, for example, show increased dextran penetrance without alterations in active zone number or receptor clustering [55].

Altered V-ATPase activity could result in altered neurotransmitter clearance. Lysosomal pH elevation due to defective V-ATPase activity may result in vesicle proteins (transporters, pumps, and SNARES) not being properly recycled or degraded. As a result, this could lead to inefficient neurotransmitter clearance efficiency, as vesicles would not be able to reload neurotransmitters correctly. Vha55 localizes to the lysosome and is responsible for proton movement [56]. This lysosomal transport is vital for recycling of various proteins and additional signaling molecules. Lysosomal transport is also needed for neurotransmitter recycling at the synapse. A subunit of V-ATPases (ductin) has been shown to localize to gap junctions of *Drosophila* during oogenesis, suggesting that V-ATPase-driven activity may regulate pathways involved in septate junction formation [57]. Additionally, transcriptomic and imaging approaches could clarify how Vha55 influences the endosomal/lysosomal system in SPG. Combining pH-sensitive reporters with live trafficking assays of junctional proteins (e.g., Neuroglian, NrxIV) would provide direct evidence for a role of Vha55 in endosomal regulation of septate junction assembly. Such studies would also connect the molecular function of V-ATPases to broader mechanisms of barrier integrity in glia.

In this manuscript, we demonstrate that glial-specific loss of miR-92 and glial-specific upregulation of Vha55 result in increased immature boutons that contain neurotransmitters but lack active zones. A homeostatic characteristic of the NMJ is the ability to generate ghost boutons upon exposure to high K^+^ hemolymph. Ataman et al. demonstrated that exposure to 90 mM K^+^ induces the addition of new ghost boutons after roughly 150 min [21]. The modifications in ghost bouton number we are seeing on our animals indicate an improper ability to regulate synaptic maturation [28]. Improperly formed septate junctions in the SPG may be impacting how the nervous system of miR-92 aberrant animals facilitate their synaptic maturation. We do not rule out additional targets downstream of miR-92 as potential regulators of synaptic development. We highlight how Vha55 functions downstream miR-92 through its rescuing of aberrant effects found upon loss of miR-92. Additional trans-glial signaling pathways such as (Wg/Wnt) may be conduits to the phenotypes we report in this study. One could hypothesize a similar mechanistic regulation based upon prior work connecting these pathways to similar developmental effects [31].

This manuscript provides a mechanistic model demonstrating *Drosophila* miR-92 in Subperineurial glia regulates Vha55, which is, in turn, required for appropriate septate junction formation and barrier function for the neuronal tissue of the NMJ. This incomplete septate junction formation coincides with an increase in immature boutons at the NMJ and subdued electrophysiological responses.

### 4.5. Human Disease

Our results show that glial miR-92 regulates NMJ development via Vha55, a mechanism with potential parallels in human disease. In schizophrenia, abnormal miR-92 expression and reduced glutamatergic synapses have been reported [58], while BBB disruption and altered ATP6V1B2 (the human homolog of Vha55) expression are also observed [59,60]. Because ATP6V1B2 dysfunction has been linked to mitochondrial abnormalities, it is possible that miR-92–Vha55 regulation reflects a conserved mechanism underlying neural pathology. Beyond Vha55, however, our microarray results point to a network of additional predicted targets that may also contribute to synaptic and barrier dysfunction. This raises the possibility that miR-92 controls a broader suite of transcripts whose dysregulation could converge on pathways disrupted in psychiatric disorders.

Although cross-species extrapolation must be cautious, these findings underscore the value of future studies in rodent and human models to determine whether miR-92-mediated regulation of V-ATPases and additional predicted targets contributes to psychiatric pathophysiology. High-resolution transcriptomic and CLIP-based analyses in these systems will be critical to define whether the downstream networks we identify in *Drosophila* are conserved in mammals.

## 5. Conclusions

The complexity of underlying biological mechanisms of aberrant neuronal conditions is becoming increasingly apparent. Dysregulated miRNAs are gaining more attention as they are found to coincide with these disease states. miR-92 is involved in synaptogenesis, synaptic function, and plasticity. MiR-92 expression is specifically required within glial tissue for normal synaptic growth and barrier functionality, and many of these functions are tied to its regulation of the target gene Vha55. Additionally, we lay the groundwork for future analysis of additional miR-92 targets playing relevant functions at the NMJ. This work further highlights how proper glial cellular function contributes to underlying neuronal development. This study indicates that miR-92 is required in *Drosophila* glial tissue for normal neuronal function and indicates a possible miRNA mechanism within glial tissue may underlie human disease pathology.

## Data Availability

The raw data supporting the conclusions of this article will be made available by the authors on request.

## Supplementary Materials

The following supporting information can be downloaded at: https://www.mdpi.com/article/10.3390/biom15091330/s1, Supplemental Table S1: Microarray dataset for miR-92 targets; Supplemental Figure S1: Immunohistochemical analysis of loss of miR-92 and upregulation of Vha55 (A.) Relative GluRIIA fluorescence intensity in miR-92a/b null mutant animals compared to W^118^ control flies. n = 10–11. (B.) Relative GluRIIA fluorescence intensity in Repo-Gal4 driven miR-92b sp animals compared to Repo-Gal4 driven sponge control files. n = 12–14. (C.) Relative fluorescence from Anti-Vha55 antibody staining at the NMJ of W^118^ and Vha55-RNAi animals. Fluorescence is significantly reduced upon pan-glial specific inhibition using Vha55-RNAi. Relative fluorescent signal was calculated using ImageJ using HRP as a benchmark, n = 10; Supplemental Figure S2: Pre- and Post-Synaptic markers are unchanged in Glial Vha55 overexpression (A.) Relative GluRIIA intensity in Repo-Gal4 driven Vha55 flies compared to Repo-Gal4 driven W^118^ control. n = 11–14. (B.) Relative Brp intensity in Repo-Gal4 driven Vha55 animals compared to Repo-Gal4 driven W^118^ control. Relative fluorescent signal was generated using anti-BRP(1:50) and calculated with ImageJ using HRP as a benchmark, n = 14–17; Supplemental Figure S3: Transmission electron microscopy (TEM) of wandering third instar larvae. (A.) Peripheral nerves of Repo-Gal4 driven VHA55 loss and miR-92 mutant animals compared to control flies. The septate junction of the subperineurial glia appears to have ‘blurry’ sections with undefined cross-hatching (white bars). (B.) TEM of the neural lamella at the outside of the peripheral nerve. The Neural lamella appears to be increased in thickness compared to control flies (red arrow). Transmission electron microscopy of peripheral nerves of miR-92a/b null mutant animals compared to control flies. The septate junction of the subperineurial glia appears to have ‘blurry’ sections with undefined cross-hatching (white arrows). The Neural lamella is increased in thickness at the peripheral edge of the nerve compared to control flies (red arrow). Images are representative of 5 images of peripheral nerves per animal from 2 animals per genotype.

## Abbreviations

BBB	Blood brain barrier
NMJ	neuromuscular junction
BRP	bruchpilot
GPCR	G-protein coupled receptor
SPG	subperineurial glia
PG	perineurial glia
WG	wrapping glia
RRP	readily releasable pool
CNS	central nervous system
PNS	peripheral nervous system
HRP	horseradish peroxidase
