# Supplementary material for: MiR-92 Controls Synaptic Development Through Glial Vha55 Regulation
Source: Biomolecules. 2025 Sep 18;15(9):1330. doi: 10.3390/biom15091330 (PMC12467677; doi:10.3390/biom15091330)
Supplement: Supplementary file 1 [file biomolecules-15-01330-s001.zip › Supplemental Table S2.pdf]

Supplemental Table S2

Abbreviations

Abbreviation	Definition
ANOVA	Analysis of variance
AP-1	Activator protein-1 (Fos/Jun transcription factor)
ATP6V1B2	Human homolog of Drosophila Vha55 (V-ATPase subunit B)
BBB	Blood–brain barrier
Brp	Bruchpilot
CNS	Central nervous system
DLG	Discs-large
DSHB	Developmental Studies Hybridoma Bank
dme	Drosophila melanogaster (prefix for Drosophila miRNAs in figures)
Dmef2-GAL4	Driver for muscle-specific expression (myocyte enhancer factor 2 promoter)
EJP	Excitatory junctional potential
FDR	False discovery rate
FITC	Fluorescein isothiocyanate
FWER	Family-wise error rate
GAL4	Galactose-responsive transcription factor 4 (yeast)
GFP	Green fluorescent protein
GluRIIA	Glutamate receptor IIA subunit

HEPES	4-(2-hydroxyethyl)-1-piperazineethanesulfonic acid
HL3.1	Hemolymph-like saline, version 3.1
HRP	Horseradish peroxidase
hsa	Homo sapiens (prefix for human miRNAs in figures)
K+	Potassium ion
LOF	Loss of function
MAS5	Microarray Suite 5.0 (Affymetrix normalization algorithm)
MATLAB	Matrix Laboratory (MathWorks software)
mEJP	Miniature excitatory junctional potential
miR / miRNA	MicroRNA
MRE	miRNA response element
NMDA	N-methyl-D-aspartate (receptor)
NMJ	Neuromuscular junction
OK6-GAL4	Driver for motor neuron–specific expression
PBS	Phosphate-buffered saline
PG	Perineurial glia
Repo-GAL4	Driver for pan-glial expression
Repo	Reversed polarity (glial marker)
RNAi	RNA interference
ROI	Region of interest
RRP	Readily releasable pool (of synaptic vesicles)
SPG	Subperineurial glia

sp	miRNA "sponge" (competitive inhibitor transgene)
SNP	Single-nucleotide polymorphism
SSR	Subsynaptic reticulum
TEM	Transmission electron microscopy
Tub-GAL4	Driver for ubiquitous expression (tubulin promoter)
UAS	Upstream activating sequence (GAL4–UAS system)
V-ATPase	Vacuolar H+-ATPase
Vha55	Vacuolar H+-ATPase 55-kDa B subunit (Drosophila)
VNC	Ventral nerve cord
WG	Wrapping glia
Wg/Wnt	Wingless/Wnt signaling pathway
