# Supplementary figures and images for: MiR-92 Controls Synaptic Development Through Glial Vha55 Regulation
Source: Biomolecules. 2025 Sep 18;15(9):1330. doi: 10.3390/biom15091330 (PMC12467677; doi:10.3390/biom15091330)

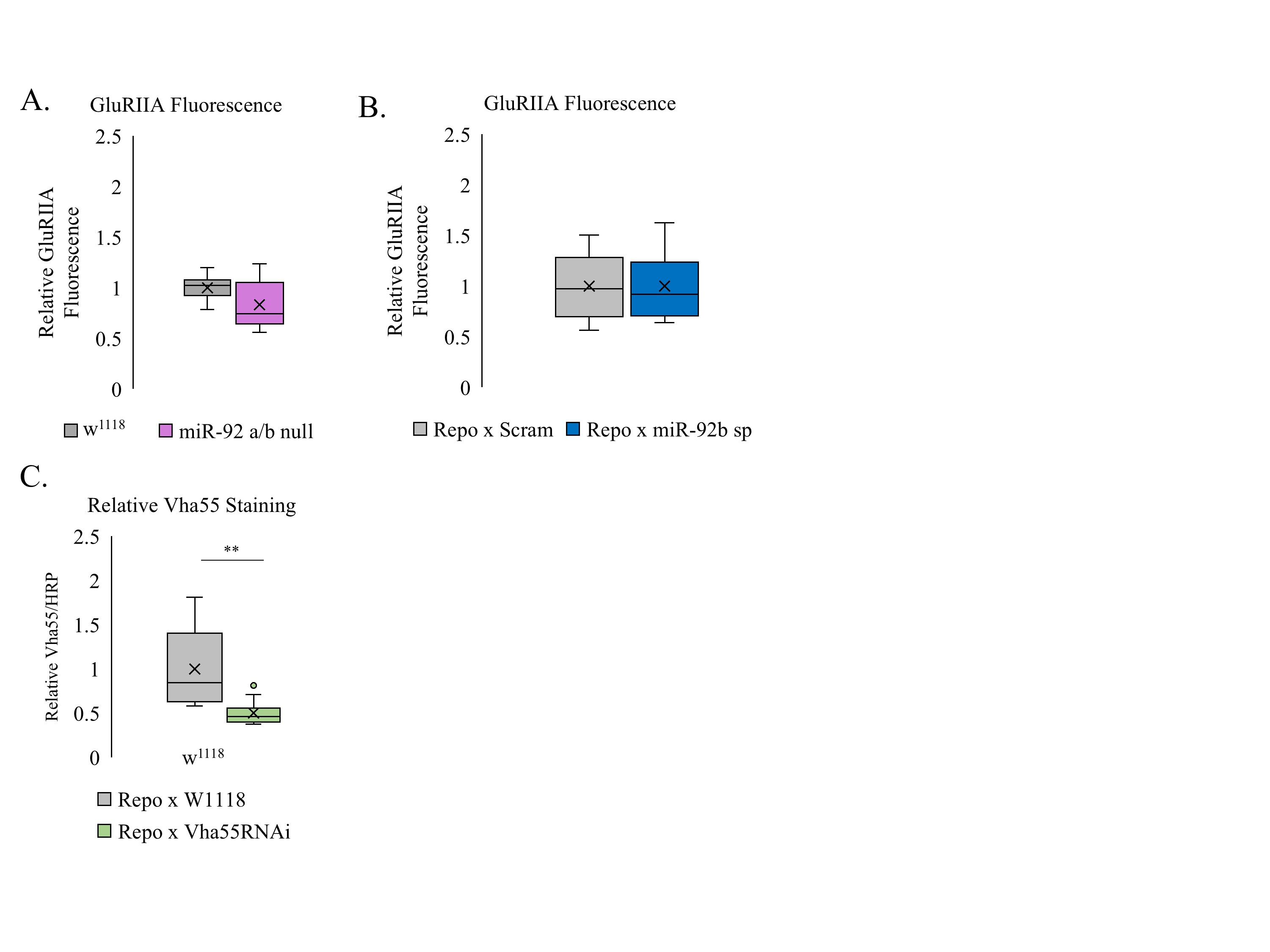

Supplement: Supplementary file 1 [file biomolecules-15-01330-s001.zip › Figure S1.jpg]

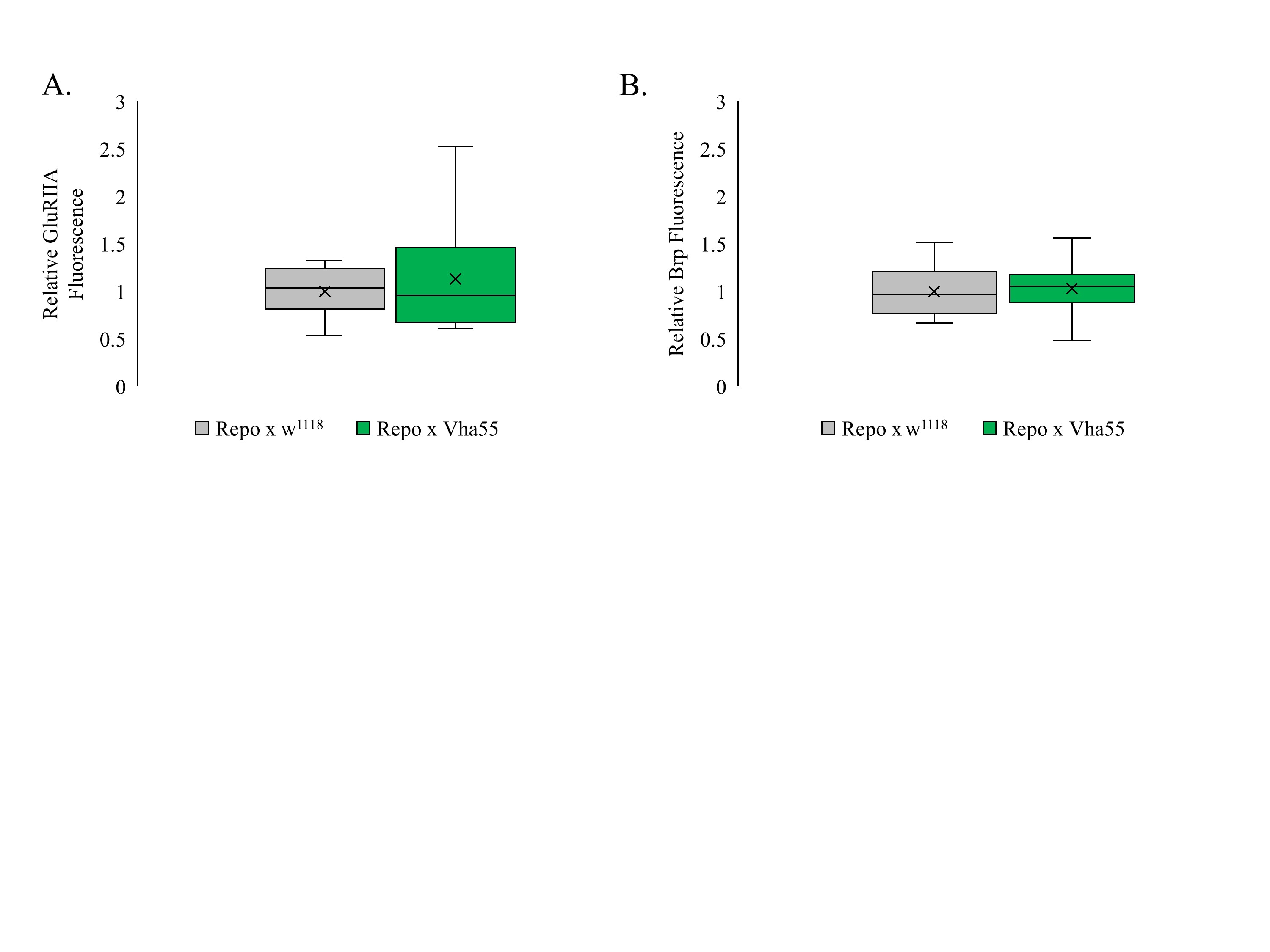

Supplement: Supplementary file 1 [file biomolecules-15-01330-s001.zip › Figure S2.jpg]

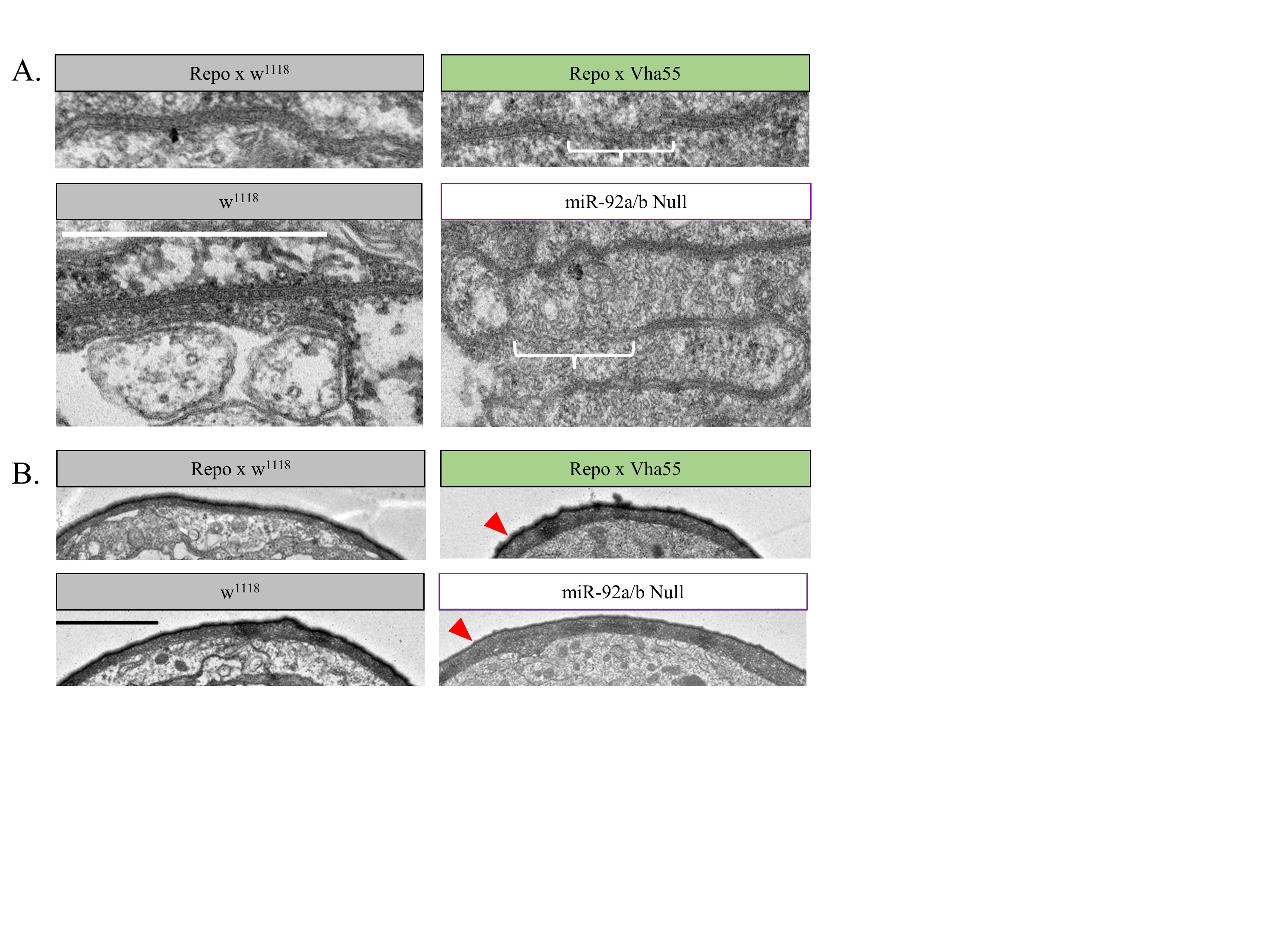

Supplement: Supplementary file 1 [file biomolecules-15-01330-s001.zip › Figure S3.jpg]
